# Amentoflavone-Enriched *Selaginella rossii* Protects against Ultraviolet- and Oxidative Stress-Induced Aging in Skin Cells

**DOI:** 10.3390/life12122106

**Published:** 2022-12-14

**Authors:** Hwa Lee, Soo-Yong Kim, Sang Woo Lee, Sehan Kwak, Hulin Li, Renzhe Piao, Ho-Yong Park, Sangho Choi, Tae-Sook Jeong

**Affiliations:** 1 Microbiome Convergence Research Center, Korea Research Institute of Bioscience and Biotechnology (KRIBB), Daejeon 34141, Republic of Korea; 2 International Biological Material Research Center, Korea Research Institute of Bioscience and Biotechnology (KRIBB), Daejeon 34141, Republic of Korea; 3 Department of Medical Biotechnology, Yeungnam University, Gyeongsan 38541, Republic of Korea; 4 Department of Agronomy, Yanbian University Agriculture College, Yanji 133000, China

**Keywords:** *Selaginella rossii*, matrix metalloproteinases, amentoflavone, skin aging, anti-wrinkle

## Abstract

Selaginellaceae plants are used in cosmetics to limit skin aging. This study is the first to investigate the anti-aging effects of *Selaginella rossii* (SR) on ultraviolet B (UVB)- and oxidative stress-induced skin cells. The 95% ethanol extract of *Selaginella rossii* (SR95E) contained much higher amounts of amentoflavone (AMF), an active compound, than other Selaginellaceae plants and was more effective in inhibiting matrix metalloproteinase (MMP)-1 expression in CCD-986sk fibroblasts. SR95E significantly decreased UVB-induced *MMP-1*, *MMP-2*, *MMP-3* and *MMP-9* expression and enhanced procollagen type I C-peptide content and mRNA expression of collagen type I alpha (*COL1A)1* and *COL1A2* in CCD-986sk fibroblasts. In HaCaT keratinocytes, SR95E treatment also dose-dependently decreased UVB-induced MMP-1 concentration and *MMP-1*, *MMP-2*, *MMP-3* and *MMP-9* mRNA expression. Moreover, SR95E treatment markedly inhibited UVB-induced c-Jun N-terminal kinase and p38 mitogen-activated protein kinase signaling and nuclear factor kappa-B signaling in HaCaT cells. Furthermore, SR95E and AMF markedly regulated the 2,2′-azobis(2-amidinopropane) dihydrochloride (AAPH)-induced expression of cellular senescence-related markers, including *p16*, *p21* and *LMNB1*, in HaCaT cells. Overall, this study indicates that SR may have potential as a functional material on preventing UVB- and AAPH-induced skin aging and wrinkles.

## 1. Introduction

The older population suffers from a variety of skin disorders such as eczema, actinic keratosis, and infectious diseases [1]. Most people over 65 years have a minimum of two skin disorders that require clinical attention [2]. Skin disorders in older individuals progress with aging of skin. There are two types of skin aging: intrinsic and extrinsic. Intrinsic aging affects all skin areas and is mainly visible in photo-protected areas [3], while intrinsic aging is characterized by thin, dry, fine wrinkles, and irregular hair growth. On the other hand, extrinsic aging affects exposed skin areas due to environmental factors such as solar radiation, smoking, and pollution. Extrinsic aging, especially photoaging, is characterized by deep and thick wrinkles, loss of elasticity, dryness, laxity, and actinic lentigines [4].

In skin aging, several factors, including ultraviolet (UV) irradiation, pollution, reactive oxygen species (ROS), telomere attrition, matrix metalloproteinase (MMP) up-regulation, and decreased collagen and elastin, alter the cellular condition of the skin, resulting in the formation of wrinkles [5]. Skin aging of the face is mainly caused by UV irradiation [6]. Photo-aging causes many structural and functional changes in skin tissue, including in the epidermis, dermis, and hypodermis. Keratinocytes are the main cell type in the epidermis, accounting for 95% of epidermal cells [7]. During skin aging, MMPs play a critical role in the formation of wrinkles by remodeling the extracellular matrix (ECM) [8]. MMPs hydrolyze ECM proteins in the dermis, including collagen and elastin [9]. In skin tissue, MMPs are secreted by keratinocytes, fibroblasts, macrophages, endothelial cells, and mast cells [8]. Epidermal keratinocytes secrete more MMPs than dermal fibroblasts in the human skin in vivo [9]. Under UV irradiation, epidermal keratinocytes exhibit decreased proliferative capacity and cell renewal [10]. The production of dermal components, such as collagen and elastin fibrils, is fragmented and reduced by photodamage [10]. Accumulated collagen fragments in the dermis decreases the mechanical forces exerted on fibroblasts. Therefore, the function of dermal fibroblasts is altered and impaired, resulting in increased production of MMPs and decreased collagen synthesis [7]. In addition, ROS loading in the epidermis is higher than that in the dermis [11]. ROS accumulation can accelerate MMP synthesis by activating mitogen-activated protein kinase (MAPK) and nuclear factor kappa-B (NF-κB) signaling [3].

*Selaginella tamariscina* (ST), a Sellaginellaceae plant, is a well-known traditional medicine for the treatment of metabolic disorders, several inflammatory diseases, and various cancers in many Asian countries [12]. In Korea, ST extract has been used in cosmetic applications such as skin hydration, whitening, and anti-wrinkle products [12]. *S. involvens* (SI) extract has been reported to possess anti-acne activity by inhibiting the inflammatory response and *Propiobacterium acnes* [13]. *Selaginella rossii* (SR) has been reported to exert anti-obesity, anti-diabetic, and anti-cancer effects [14,15]. SR, known as Gu-sil-sa-ri in Korea and Lu jiao juan bai in China, has been traditionally used to treat cancer and prevent bleeding [16]. *Selaginella* species, including ST, SI and *S. pulvinata,* have been reported to contain various selaginellins and biflavonoids such as amentoflavone (AMF), sequoiaflavone, and hinokiflavone [12,17,18,19]. Among them, AMF has been reported to confer anti-obesity, anti-diabetic, anti-tumor and anti-wrinkle effects [14,20,21,22]. However, SR has not been reported to exert anti-wrinkle effects. Therefore, the aim of this study was to investigate the effect of SR extract on skin fibroblasts and keratinocytes, and to further elucidate whether AMF serves as an active compound.

## 2. Materials and Methods

### 2.1. Preparation of SR Extracts

The dried aerial part of *Selagenella rossii* (Barker) Warb (SR) were obtained from Yanbian University (Yanji, China) delivered by the International Biological Material Research Center (IBMRC, Daejeon, Republic of Korea) of the Korea Research Institute of Bioscience and Biotechnology (KRIBB, Daejeon, Republic of Korea). The 1 g dried SR powders were extracted using 10 mL 70% EtOH, 95% EtOH or MeOH at room temperature for 48 h, respectively. The samples of 70% EtOH extract of SR (SR70E), 95% EtOH extract of SR (SR95E), and MeOH extract of SR (SRM) were concentrated under reduced pressure to yield brown residues 158 mg, 94 mg and 102 mg, respectively. The MeOH extracts of ST (STM) and SI (SIM) were obtained from the IBMRC of KRIBB.

### 2.2. Phytochemical Analysis of SR Extracts

SR95E and SR70E were subjected to qualitative phytochemical analysis for the presence or absence of alkaloids, flavonoids, glycosides, phenolics, tannins and phytosterols, according to preliminary qualitative [23], EMD Chemical catalog of TLC visualization reagents (https://emdchemicals.lookchem.com/ (accessed on 30 November, 2022)), and high-performance liquid chromatography (HPLC) analyses.

### 2.3. Cell Culture and Detection of Cell Viability

The CCD-986sk human skin fibroblasts were purchased from the Korean Cell Line Bank (Seoul, Republic of Korea) and cultured in Iscove’s Modified Dulbecco’s Medium (IMDM; Welgene Inc., Gyeongsan, Republic of Korea) containing 10% fetal bovine serum (FBS; Life Technologies Corporation, Grand Island, NY, USA), 1% Penicillin-Streptomycin solution (Hyclone, Logan, UT, USA) at 37 °C in a humidified incubator with air containing 5% CO_2_. The HaCaT human keratinocytes (Cell Line Service, Eppelheim, Germany) were cultured in Dulbecco’s Modified Eagle Medium (DMEM; Life Technologies Corporation, Grand Island, NY, USA) containing 2 mM L-glutamine (Life Technologies Corporation, Grand Island, NY, USA), 10% FBS, and 1% Penicillin-Streptomycin solution at 37 °C in a humidified incubator with air containing 5% CO_2_.

CCD-986sk fibroblasts or HaCaT cells were plated in 96-well plates and after adhesion to plates treated with extracts or AMF for 24 h. UVB-irradiation was conducted using a UVB lamp (Sankyo-Danki, Hiratsuka, Japan) which emits wavelengths between 280 nm to 360 nm and peaked at 305–310 nm. Cell viability was detected using the D-Plus™ CCK kit (Donginls, Daejeon, Republic of Korea).

### 2.4. Measurement of MMP-1 and Procollagen Secretion Levels

MMP-1 and procollagen secretion levels were measured as previously reported [24]. CCD-986sk or HaCaT cells were irradiated with UVB and treated with SRE or AMF for 24 h. MMP-1 levels in CCD-986sk or HaCaT cells were measured using the MMP-1 ELISA kit (Abcam, Cambridge, MA, USA). Concentration of PIP in CCD-986sk cells was detected by procollagen type I C-peptide (PIP) EIA kit (Takara Bio Inc., Shiga, Japan).

### 2.5. Detection of Reactive Oxygen Species (ROS)

HaCaT cells were plated in 96-well plates and treated with the SRE or AMF for 2 h. After 24 h of UVB-irradiation, cells were incubated with 20 μM 2′,7′-dichloro-fluorescin diacetate (Thermo Fisher Scientific, Waltham, MA, USA) for 60 min. Fluorescence intensities were measured using a spectrofluorometer (Wallac 1420; Perkin-Elmer, Turku, Finland) at 485/535 nm (Ex/Em).

### 2.6. Quantitative Real-Time RT-PCR

SRE or AMF treated cells were prepared for total RNA using TRI solution (Ambion, Carlsbad, CA, USA). Total RNA was used to synthesize cDNA using a High-capacity cDNA Reverse Transcription Kit (Applied Biosystems, Foster City, CA, USA). For detection of mRNA expression level, a quantitative Real-Time PCR system (Life Technologies, Grand Island, NY, USA) with Power SYBR Green PCR Master Mix (Applied Biosystems, Woolston, Warrington, UK) were used to conduct Real-Time RT-PCR. The used primers are shown in Table S1.

### 2.7. Western Blot

For detection of MAPKs and NF-κB signaling pathways, anti-p- extracellular signal-regulated kinase (ERK), anti-ERK, anti-p-c-Jun N-terminal kinase (JNK), anti-p-p38, anti-p-NF-κB p65, and anti-NF-κB p65 antibodies (Cell Signaling Technology, Danvers, MA, USA) and anti-JNK and anti-p38 antibodies (Santa Cruz Biotechnology, Dallas, TX, USA) were used. Protein expression was determined using a Chemiluminescent HRP Substrate (Merck Millipore, Burlington, MA, USA) and a LAS-4000 luminescent-image reader (Fuji Photo Film, Tokyo, Japan). Image MultiGauge (Fuji Photo Film, Tokyo, Japan) was used to analyze immunoreactive signals.

### 2.8. Statistical Analysis

Values of results were given as the means ± standard deviations (SD) and the significant differences between the control and treatment groups were analyzed using Student’s *t*-tests. A *p*-value < 0.05 was considered significant.

## 3. Results

### 3.1. Selaginellaceae Inhibited MMP-1 Expression in CCD-986sk Fibroblasts

**Figure 1:**
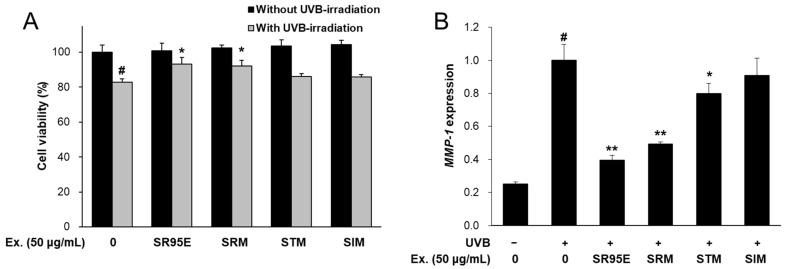
Cell viability and *MMP-1* expression of Selaginellaceae extracts in CCD-986sk cells. (**A**) Cells were treated with each extract (50 μg/mL) for 24 h with or without UVB-irradiation. Cell viability was measured by CCK kit (Donginls, Daejeon, Republic of Korea). (**B**) CCD-986sk cells were treated with the extracts of Selaginellaceae (50 μg/mL) and UVB-irradiation. After 24 h, the mRNA levels were measured by real-time qRT-PCR and normalized using *ACTIN* as a reference gene. Values are presented as means ± SD. ^#^ *p* < 0.01 vs. cells treated with media only; * *p* < 0.05, ** *p* < 0.01 vs. cells treated with UV only. SR95E, *Selagenella rossii* 95% ethanol extract; SRM, *Selagenella rossii* methanol extract; STM, *Selaginella tamariscina* methanol extract; SIM, *Selaginella involvens* methanol extract; Ex, extract; SD, standard deviations.

The potential cytotoxicity of Selaginellaceae plants, including ST, SI and SR, were evaluated in CCD-986sk fibroblasts. After treatment with 50 μg/mL of each extract, none were toxic to CCD-986sk fibroblasts under normal conditions without UVB irradiation (Figure 1A). Under UVB irradiation, the viability of the fibroblasts decreased significantly to 82.9%. However, treatment with SR95E or SRM, but not the STM or SIM, markedly restored UVB-induced cell cytotoxicity (Figure 1A). SR extracts exhibited more potent activity on *MMP-1* inhibition than the other *Selaginella* species (Figure 1B). These results correlate with the AMF contents of the Selaginellaceae extracts [14]. Hence, this study focused on the anti-wrinkle effects of SR against UVB-exposed fibroblasts and keratinocytes.

### 3.2. Phytochemical Components of SR Extracts

The phytochmical and HPLC profiles analyses showed that SR95E and SR70E illustrated the presence of alkaloids, flavonoids, glycosides, phenolics and pheophorbides (products of chlorophyll breakdown). Among the 11 species of Selaginella, biflavonoid content was high in *S. sinensis*, *S. davidii* and *S. mollendorfii* (ranging from 1.0 to 1.1%) [25]. Compared with other Selaginellaceae species such as ST and SI, SR95E and SR70E contained markedly higher AMF [14]. The AMF contents of SR95E and SR70E were 66.6 mg AMF/g extract and 46.8 mg AMF/g extract, respectively, as quantified by HPLC analysis (Figure S1). The analytical HPLC profile showed that SR95E contained a large number of its natural derivatives and different flavonoid glycosides in addition to AMF (Figure S2) [25].

### 3.3. SR Inhibited MMP-1 Secretion and MMP Expression in CCD-986sk Fibroblasts

**Figure 2:**
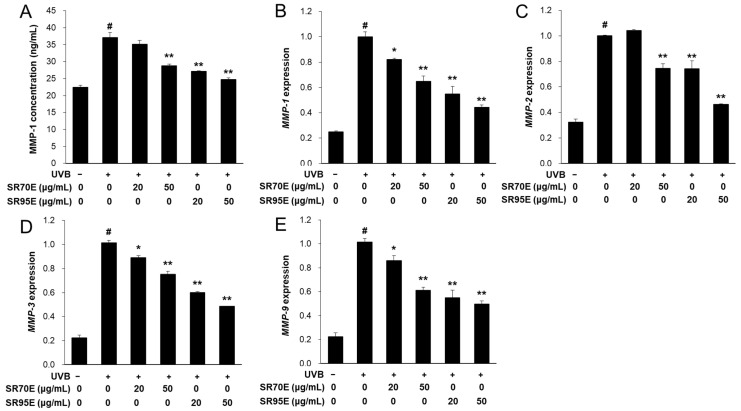
Effect of the SR extracts on matrix metalloproteinase (MMP)-1 concentration and MMPs expression in UVB-irradiated CCD-986sk fibroblasts. Cells were treated with SRE extracts and irradiated to UVB for 24 h. (**A**) MMP-1 concentrations were measured from cell culture medium. (**B**–**E**) The mRNA expressions of *MMP-1*, *MMP-2*, *MMP-3* and *MMP-9* were measured by real-time RT-PCR and normalized against those of *ACTIN* as reference gene. UVB − indicates treatment without UVB-irradiation and UVB + indicates treatment with UVB-irradiation. Data was presented as means ± SD (*n* = 3). ^#^ *p* < 0.01 vs. cells not irradiated; * *p* < 0.05, ** *p* < 0.01 vs. cells with UVB-irradiation only. SR70E, *Selagenella rossii* 70% ethanol extract.

Next, MMP-1 concentration and MMP expression levels were measured to determine the anti-wrinkle effects of SR95E and SR70E on UVB-irradiated CCD-986sk fibroblasts. Treatment with 50 μM SR70E or SR95E significantly reduced UVB-induced MMP-1 secretion by 57.1% and 84.4%, respectively (Figure 2A). In addition, the UVB-induced mRNA expression of *MMP-1*, *MMP-2*, *MMP-3* and *MMP-9* was significantly reduced by treatment with SR70E and SR95E (Figure 2B–E). SR95E exhibited much higher AMF content than SR70E, as quantified by HPLC analysis. These results confirmed that the inhibitory effects of MMPs expression depended on the AMF contents of the SR extracts. Thus, SRE can effectively inhibit skin wrinkle formation by inhibiting UVB-induced MMP expression in CCD-986sk cells.

### 3.4. SR Enhanced Procollagen Expression in CCD-986sk Fibroblasts

**Figure 3:**
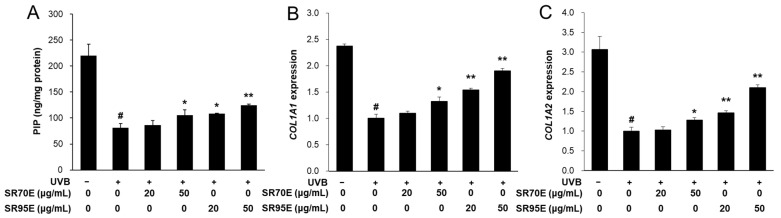
Effect of SR extracts on procollagen type I C-peptide (PIP) concentration and mRNA expression in UVB-induced CCD-986sk cells. Cells were treated with SR70E or SR95E and irradiated to UVB for 24 h. (**A**) PIP concentrations were measured from cell culture medium. (**B**,**C**) The mRNA expression of *COL1A1* and *COL1A2* were measured by real-time RT-PCR and normalized against those of *ACTIN* as reference gene. Data was presented as means ± SD (*n* = 3). ^#^ *p* < 0.01 vs. cells not irradiated; * *p* < 0.05, ** *p* < 0.01 vs. cells with UVB-irradiation only.

Skin fibroblast-synthesized procollagen is a major component in the formation of collagen fibrils in the intracellular matrix. The PIP production in UVB-irradiated CCD-986sk cells was detected to determine whether SR extract treatment could regulate procollagen synthesis. UVB irradiation significantly decreased PIP levels. However, treatment with 50 µM SR70E or SR95E significantly increased PIP concentrations compared to UVB-irradiated CCD-986sk cells (Figure 3A). Further, the mRNA expression levels of collagen type I alpha (*COL1A)1* and *COL1A2* genes related to procollagen synthesis were significantly increased by SR70E or SR95E treatment (Figure 3B,C). In addition, SR95E was more effective in promoting procollagen synthesis than SR70E. These results suggest that SR extract may exert anti-wrinkle effects by enhancing procollagen synthesis in skin fibroblasts.

### 3.5. SR Inhibited MMP-1 Secretion and MMP Expression in HaCaT Keratinocytes

**Figure 4:**
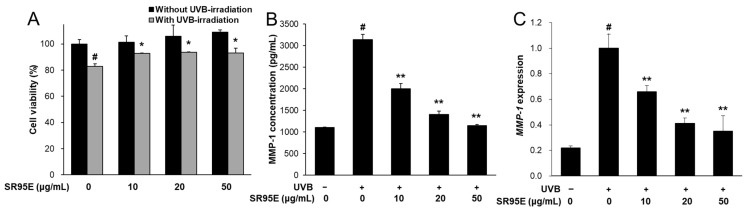

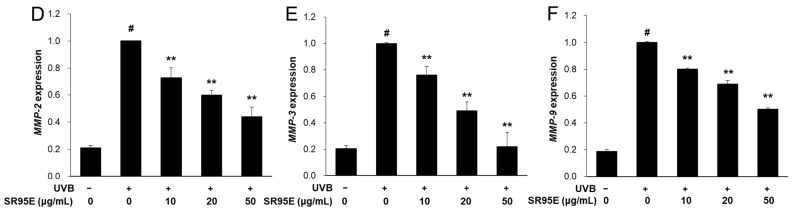
Effect of SR on MMPs expression in UVB-induced HaCaT keratinocytes. (**A**) Cells were treated with SR95E (10, 20 and 50 μg/mL) for 24 h with or without UVB-irradiation. For detection of MMPs expression, cells were treated with SR95E extracts and irradiated to UVB for 16 h. (**B**) MMP-1 concentrations were measured from cell culture medium. (**C**–**F**) The mRNA expression of *MMP-1*, *MMP-2*, *MMP-3* and *MMP-9* were measured by real-time RT-PCR and normalized against those of *ACTIN* as reference gene. Data was presented as means ± SD (*n* = 3). ^#^ *p* < 0.01 vs. cells not irradiated; * *p* < 0.05, ** *p* < 0.01 vs. cells with UVB-irradiation only.

Under UV irradiation, epidermal keratinocytes are a major source of MMP production in human skin. Therefore, the MMP-inhibitory activity of SR95E was determined in HaCaT keratinocytes. SR95E reversed UVB-induced cell damage at concentrations ranging from 10 to 50 µg/mL (Figure 4A). Treatment with SR95E significantly reduced UVB-induced MMP-1 secretion in a dose-dependent manner (Figure 4B). In addition, the mRNA expression levels of *MMP-1*, *MMP-2*, *MMP-3* and *MMP-9* were significantly reduced by SR95E treatment in UVB-treated HaCaT cells (Figure 4C–F). These results indicate that SR95E inhibits UVB-induced MMP expression in HaCaT keratinocytes.

### 3.6. SR Regulated MAPK and NF-κB Signaling in HaCaT Keratinocytes

**Figure 5:**
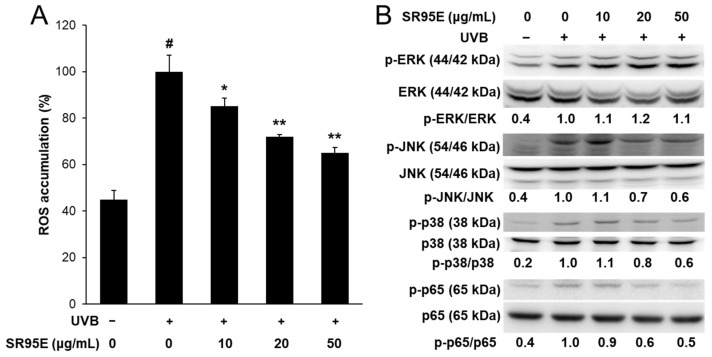
Effect of SRE on reactive oxygen species (ROS) accumulation and mitogen-activated protein kinase (MAPK)/NF-κB signaling in UVB-induced HaCaT keratinocytes. (**A**) Cells were treated with SRE and irradiated to UVB. After 24 h, ROS accumulation was determined using 2′,7′-dichloro-fluorescin diacetate. Data was presented as means ± SD (*n* = 3). ^#^ *p* < 0.01 vs. cells not irradiated; * *p* < 0.05, ** *p* < 0.01 vs. cells with UVB-irradiation only. (**B**) The total and phosphorylation protein levels of ERK, JNK, p38, and NF-κB p65 were detected by Western blot.

Under UVB irradiation, increased cellular ROS levels enhanced MMP expression through the activation of the MAPK and NF-κB signaling pathways. Therefore, we determined the effects of SR95E on ROS levels and the MAPK/NF-κB signaling pathways. UVB-induced ROS levels decreased significantly in a dose-dependent manner by SR95E treatment (Figure 5A). Furthermore, UVB-induced phosphorylation levels of JNK and p38 were markedly decreased by SR95E treatment, but not by ERK (Figure 5B). In addition, the phosphorylation of NF-κB p65 markedly increased after UVB irradiation. However, SR95E treatment decreased the phosphorylation of NF-κB p65 (Figure 5B). These results suggest that SR95E can inhibit UVB-induced MMP expression by inhibiting ROS accumulation and the JNK MAPK, p38 MAPK, and NF-κB p65 signaling pathways.

### 3.7. AMF Inhibited UVB-Induced Skin Aging in HaCaT Keratinocytes

**Figure 6:**
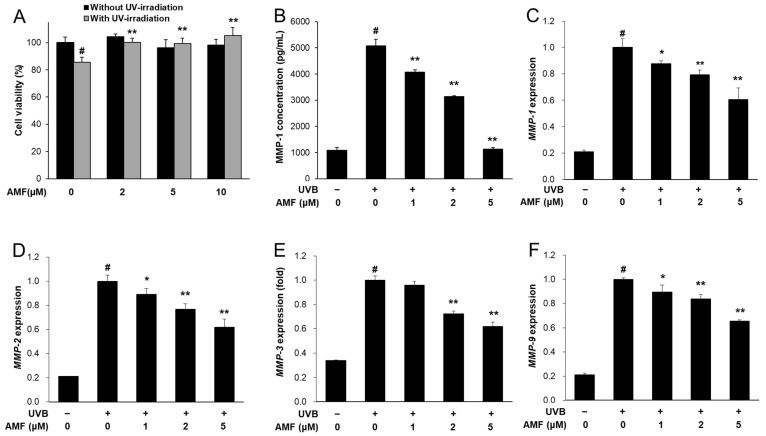
Effect of amentoflavone (AMF) on MMPs expression in UVB-induced HaCaT keratinocytes. (**A**) Cells were treated with AMF (2, 5 and 10 μM) for 24 h with or without UVB-irradiation. For detection of MMPs expression, cells were treated with AMF (1, 2 and 5 µM) and irradiated to UVB for 16 h. (**B**) MMP-1 concentrations were measured from cell culture medium. (**C**–**F**) The mRNA expression of *MMP-1*, *MMP-2, MMP-3* and *MMP-9* were measured by real-time RT-PCR and normalized against those of ACTIN as reference gene. Data was presented as means ± SD (*n* = 3). ^#^ *p* < 0.01 vs. cells not irradiated; * *p* < 0.05, ** *p* < 0.01 vs. cells with UVB-irradiation only.

**Figure 7:**
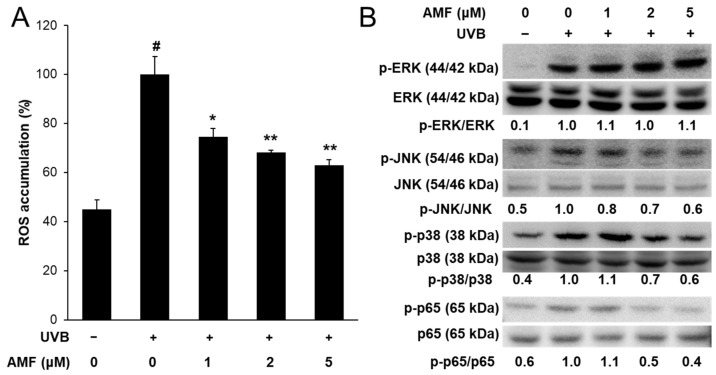
Effect of AMF on reactive oxygen species (ROS) accumulation and mitogen-activated protein kinase (MAPK)/NF-κB signaling in UVB-induced HaCaT keratinocytes. (**A**) Cells were treated with AMF and irradiated to UVB. After 24 h, ROS accumulation was determined using 2′,7′-dichloro-fluorescin diacetate. Data was presented as means ± SD (*n* = 3). ^#^ *p* < 0.01 vs. cells not irradiated; * *p* < 0.05, ** *p* < 0.01 vs. cells with UVB-irradiation only. (**B**) The total and phosphorylation protein levels of ERK, JNK, p38 and NF-κB p65 were detected by Western blot.

AMF is the main component of SRE, as shown in Figure S1. Therefore, the effect of AMF on MMP expression was determined to confirm whether AMF serves as an active compound in SRE. AMF reversed UVB-induced cell damage at concentrations ranging from 2 to 10 µM (Figure 6A). AMF treatment significantly reduced UVB-induced MMP-1 secretion in a dose-dependent manner (Figure 6B). In addition, the mRNA expression levels of *MMP-1*, *MMP-2*, *MMP-3* and *MMP-9* were significantly reduced by AMF treatment in UVB-treated HaCaT cells (Figure 6C–F). Furthermore, AMF treatment significantly decreased UVB-induced ROS accumulation in a dose-dependent manner (Figure 7A). AMF treatment decreased the phosphorylation of JNK and p38 MAPK, but not that of ERK (Figure 7B). UVB-induced phosphorylation of NF-κB p65 was markedly decreased by AMF treatment (Figure 7B). These results indicate that AMF may serve as an active compound of SRE in inhibiting UVB-induced MMP expression in HaCaT keratinocytes.

### 3.8. SR and AMF Protected against AAPH-Induced Senescence in HaCaT Keratinocytes

**Figure 8:**
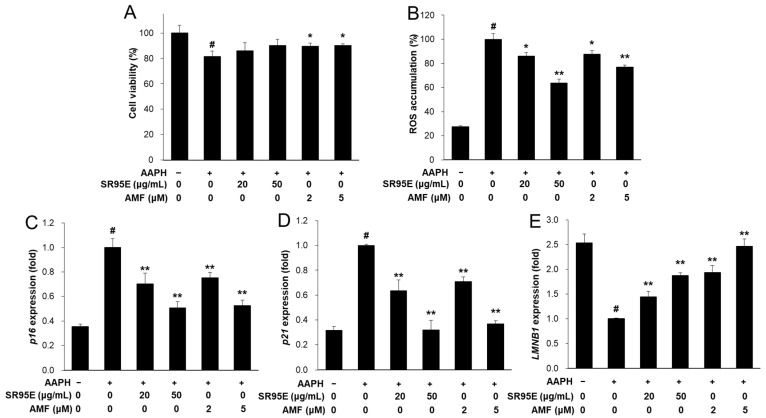
Effect of SR and AMF in AAPH-induced senescent HaCaT keratinocytes. (**A**) Cells were treated with SR95E or AMF for 24 h with 2 mM AAPH. For detection of MMPs expression, cells were treated with SRE or AMF and irradiated to UVB for 24 h. (**B**) Cells were treated with SRE or AMF and induced with AAPH. After 24 h, ROS accumulation was determined using 2′,7′-dichloro-fluorescin diacetate. (**C**–**E**) The mRNA expression of *p65*, *p21* and *LMNB1* were measured by real-time RT-PCR and normalized against those of *ACTIN* as reference gene. Data was presented as means ± SD (*n* = 3). ^#^ *p* < 0.01 vs. cells not induced; * *p* < 0.05, ** *p* < 0.01 vs. cells with AAPH-induced only.

Senescent cells accumulate in aged skin tissues and cause skin aging. Cellular senescence-related markers were investigated in 2,2-azobis(2-amidinopropane) dihydrochloride (AAPH)-treated HaCaT cells. Prior to the detection of cellular senescence markers, the viability of AAPH-treated HaCaT cells was measured. The viability of the AAPH-treated HaCaT cells was significantly decreased to 81.4%. Treatment with AMF, but not SR95E, slightly restored AAPH-induced cytotoxicity (Figure 8A). In addition, treatment with SR95E or AMF significantly decreased AAPH-induced ROS accumulation in HaCaT keratinocytes (Figure 8B). Furthermore, AAPH-induced mRNA expression of cellular senescence-related cell cycle regulators, including *p16* and *p21*, was significantly reduced by treatment with SR95E or AMF (Figure 8C,D). Nuclear lamin B1 (*LMNB1*) expression was decreased in AAPH-treated HaCaT cells. However, treatment with SR95E or AMF significantly upregulated *LMNB1* expression (Figure 8E). These results suggest that SR95E and its active compound AMF can improve oxidative stress-induced cellular senescence.

## 4. Discussion

Although many Selaginellaceae plants including ST and SI have been used as traditional medicines in Asia, little is known about the bioactivities of SR. In a previous study, we reported that SRE and SRM were much more enriched in AMF than the methanol extracts of other Selaginellaceae plants, ST and SI [14]. In addition, in our previous study we also found that AMF-enriched SR extracts inhibited high-fat diet-induced obesity and hyperglycemia by suppressing intestinal lipid absorption [14]. During the progression of skin photoaging, AMF inhibits MMP-1 expression through the inhibition of ERK/activator protein (AP)-1 activation (but not via JNK and p38) in normal human fibroblasts [22]. In addition, AMF protects against nuclear aberrations in UVB-induced human fibroblasts [26]. Our results confirmed that the MMPs-inhibitory activities of SR70E and SR95E were dependent on their AMF content. Both SR70E and SR95E inhibited MMP expression and increased collagen synthesis in CCD-986sk fibroblasts.

*Selaginella* species are rich sources of bioflavonoids. Among the bioflavonoids identified from ST, AMF (3′,8″-biapigenin), and sumaflavone (6″-*O*-hydroxy AMF) showed significant inhibitory activity against MMP-1 in UV-irradiated human fibroblasts, while 2′,8″-biapigenin, robustaflavone (3′,6″-biapigenin) and taiwaniaflavone (6-methy-7,4′-di-*O*-methyl AMF) had no significant effects. These differences in MMP-1 inhibitory activity are likely due to structural differences, particularly those related to the location of the C–C bond and number of hydroxyl groups in the flavonoid skeleton, which can cause potent nitric oxide blocking effects [27]. After oral administration, AMF was rapidly metabolized into 39 products via oxidation, internal hydrolysis, hydrogenation, methylation, sulfation, glucuronidation, glucosylation, *O*-aminomethylation, and degradation in rats. Moreover, *O*-aminomethylation and glucosylation are metabolic pathways unique to AMF [28]. AMF metabolites should be further investigated to provide more information on the safety and efficacy of SR extracts with high AMF content. The bioavailability of AMF with oral administration (0.04% ± 0.01% at 300 mg/kg) was much lower than that of intraperitoneal injection (77.4% ± 28.0% at 10 mg/kg) [29]. Pheophorbides, especially pheophorbode a and pyropheophorbide a, exert anti-wrinkle effects in UVB-induced fibroblasts by suppressing MMPs expression and MAPK/NF-κB signaling [24]. The pheophobide contents of SR aerial extracts were not high.

In aged skin, changes in the ECM composition, including fragmented collagen and elastin fibrils, can affect the phenotypes of skin cells [5]. Fibroblasts in aged skin are characterized by a collapsed cytoplasm and lack of connections to surrounding collagen fibrils, with decreased cell surface area. The fibroblasts in age-associated dermal microenvironments produce ROS, MMPs, and cysteine-rich proteins [30,31]. Moreover, there is a higher load of age-related biomolecules, including ROS, MMPs, and cysteine- rich proteins, in the epidermis compared to the dermis [9,32,33]. Among MMPs, only MMP-1 can degrade intact collagen fibrils, whereas other types of MMPs, including MMP-2, MMP-3 and MMP-9, can further decompose degraded collagen fragments [7]. SR extracts effectively suppressed UVB-induced *MMP-1*, *MMP-2*, *MMP-3* and *MMP-9* expression and MMP-1 secretion in CCD-986sk fibroblasts and HaCaT keratinocytes.

UV-induced ROS can upregulate MMP expression through activation of MAPK, AP-1, nuclear factor erythroid 2-related factor 2 (Nrf2), and NF-κB signaling in keratinocytes, melanocytes, and fibroblasts [34]. MAPK signaling includes the ERK, JNK and p38 signaling pathways. Mitogenic stress activates the ERK MAPK pathway, whereas cellular stress activates the JNK and p38 MAPK pathways [35]. UVB irradiation leads to activation of the JNK and p38 MAPK pathways, but there are several factors which may lead to the activation of ERK MAPK dependent on cell conditions [36,37,38]. Under UVB irradiation, normal human keratinocytes exhibit suppressed activation of ERK MAPK, whereas HaCaT cells exhibit enhanced activation of ERK MAPK [36]. In this study, UVB irradiation also induced all of the above MAPKs, including ERK, JNK and p38, in HaCaT cells. However, SR95E and AMF dose-dependently suppressed UVB-induced ROS accumulation by inhibiting JNK and p38 MAPK activation, but not ERK MAPK activation signaling in HaCaT keratinocytes. Keratinocytes are the most abundant cells in the epidermis, and UV-induced NF-κB signaling is more rapid in keratinocytes than in human dermal fibroblasts [39]. In UVB-irradiated keratinocytes, activation of NF-κB signaling is dependent on activation of p38 MAPK [40,41]. In this study, SR95E and AMF markedly inhibited the UVB-induced activation of p38 MAPK and NF-κB signaling in HaCaT keratinocytes.

In aged skin, senescent cells promote skin aging. Senescent cells display various changes, such as irregular shape, loss of lamin B1 with compromised nuclear integrity, and increased cytoplasmic chromatin fragments [42]. Cellular senescence is accompanied by decreased proliferation, resistance to apoptosis, and altered gene expression [43]. Altered gene expression in senescent cells includes alteration of genes related to cell cycle regulation, inflammation, the cytoskeleton, and metabolism [44,45]. Some well-known cell cycle regulatory genes include *p53*, *p16* and *p21* [44,46,47]. Under conditions such as UV irradiation and oxidative stress, p53 plays a critical role in the cell cycle arrest of senescent cells [48]. p53 acts as a transcription factor and p21 is the target gene of p53. Activated p21 inhibits cyclin-dependent kinases in cell cycle arrest at the G1/S or G2/S phase [49,50]. In addition, p16 induces the accumulation of the phosphorylated retinoblastoma protein (pRB) to stop cell proliferation [51]. Briefly, senescence growth arrest is mainly controlled by p53/p21 and p16/pRB signaling. SR95E and its compound AMF increased AAPH-induced *LMNB1* expression and suppressed that of AAPH-induced cell cycle regulators including *p21* and *p16*. This study is the first to indicate the anti-senescence effects of SR and AMF. These results indicate that SR can also prevent cellular senescence in oxidative stress-induced keratinocytes.

In summary, in this study, the anti-aging effects of *Selaginellaceae* plants, including ST, SI and SR were comparatively evaluated. Among them, SR was the most effective for inhibiting UVB-induced MMP-1 expression and exhibited the highest AMF content. Both SR70E and SR95E exerted anti-wrinkle effects by suppressing MMP-1 secretion and upregulating procollagen synthesis in UVB-induced CCD-986sk fibroblasts. The anti-wrinkle activity of SR95E was also confirmed in UVB-irradiated HaCaT keratinocytes. SR95E decreased MMP-1 secretion and the expression of *MMP-1*, *MMP-2*, *MMP-3* and *MMP-9* by suppressing the JNK/p38 MAPKs and NF-κB signaling in a dose-dependent manner. The anti-wrinkle activity of SRE is mainly attributed to its main component, AMF. In addition, SR95E and AMF effectively suppressed AAPH-induced cellular senescence by suppressing *p21* and *p16* expression and enhancing *LMNB1* expression. Therefore, these findings indicate that SR has the potential to be used in functional cosmetics to prevent skin aging and wrinkles.

## Data Availability

Data are included in the article or Supplementary Materials.

## Supplementary Materials

The following supporting information can be downloaded at: https://www.mdpi.com/article/10.3390/life12122106/s1; Figure S1: HPLC profiles of 70% ethanol extract of SR (SR70E), 95% ethanol extract of SR (SR95E), and amentoflavone (AMF) detected at 225 nm; Figure S2: HPLC profiles of SR95E and UV spectra of its main phytochemical components detected at 225 nm. Table S1: Primer sequences used for qRT-PCR.
