# Supplementary material for: Amentoflavone-Enriched Selaginella rossii Protects against Ultraviolet- and Oxidative Stress-Induced Aging in Skin Cells
Source: Life (Basel). 2022 Dec 14;12(12):2106. doi: 10.3390/life12122106 (PMC9787533; doi:10.3390/life12122106)
Supplement: Supplementary file 1 [file life-12-02106-s001.zip › life-2044538-supplementary.pdf]

Amentoflavone-Enriched *Selaginella rossii* Protects Against Ultraviolet- and Oxidative Stress-Induced Aging in Skin Cells

Hwa Lee [1,†], Soo-Yong Kim [2], Sang Woo Lee [2], Sehan Kwak [1,3], Hulin Li [4], Renzhe Piao [4], Ho-Yong Park [1], Sangho Choi [2,*], and Tae-Sook Jeong [1,*]

[1] Microbiome Convergence Research Center, Korea Research Institute of Bioscience and Biotechnology (KRIBB), Daejeon 34141, Republic of Korea

[2] International Biological Material Research Center, KRIBB, Daejeon 34141, Republic of Korea

[3] Department of Medical Biotechnology, Yeungnam University, Gyeongsan 38541, Republic of Korea
[4] Department of Agronomy, Yanbian University Agriculture College, Yanji 133000, China

* Correspondence: decoy0@kribb.re.kr (S.C.); tsjeong@kribb.re.kr (T.-S.J.); Tel.: +82-42-879-8340 (S.C.); +82-42-860-4558(T.-S.J.); Fax: +82-42-861-8349 (S.C.); +82-42-861-2675 (T.-S.J.)
† Earlier known as Hua Li.

1. Amentoflavone (AMF) content and phytochemical analysis in *Selaginella rossii* (SR) extracts

High-performance liquid chromatography (HPLC) Analysis of SR70E and SR95E

The components of 70% and 95% ethanol extracts of SR aerial (SR70E and SR95E) were analyzed using a HPLC-diode array detector (HPLC-DAD) system (Shimadzu Corp., Tokyo, Japan). Extracts were separated on a Brownlee SPP C18 column (4.6 × 50 mm, 2.7 μm; Perkin Elmer, Inc, Waltham, MA, USA). The compositions of mobile phase are 0.1% acetic acid in water (mobile phase A) and acetonitrile (mobile phase B). The linear gradient elution program was as follows: 5–50% B at 0–15 min, 50–100% B at 15–20 min, 100% B at 20–25 min, 100–5% B at 25–27 min, and 5% B at 27–30 min. The absorbance of the HPLC profile was 225 nm, and the flow rate was 1.8 mL/min. Amentoflavone (AMF) (Biopurify Phytochemicals Ltd., Chengdu, China) was used as an external standard. The purity of AMF was 98.0%.

The AMF contents of SR70E and SR95E were 46.8 mg AMF/g extract and 66.6 mg AMF/g extract, respectively (Figure S1).

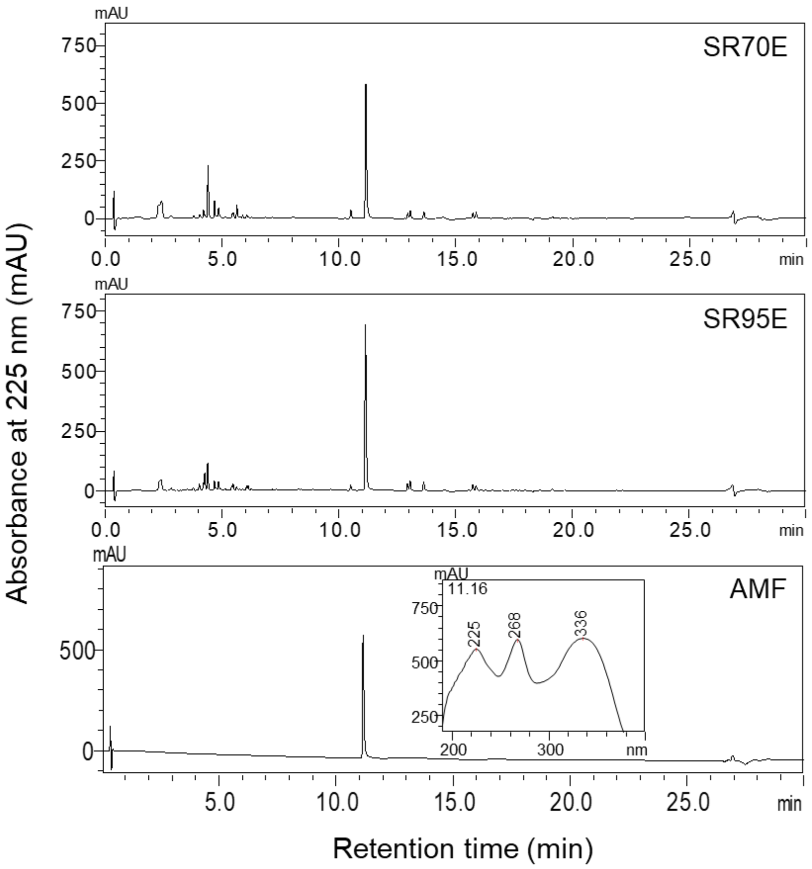

Figure S1. HPLC profiles of 70% ethanol extract of SR (SR70E), 95% ethanol extract of SR (SR95E), and amentoflavone (AMF) detected at 225 nm. Concentration of samples: SR70E (5 mg/mL); SR95E (5 mg/mL); AMF (250 μg/mL). Injection volume 5 μl.

The analytical HPLC profile showed that SR95E contained a large number of its natural derivatives (peaks 8-13) and different flavonoid glycosides in addition to AMF (Figure S2) [25].

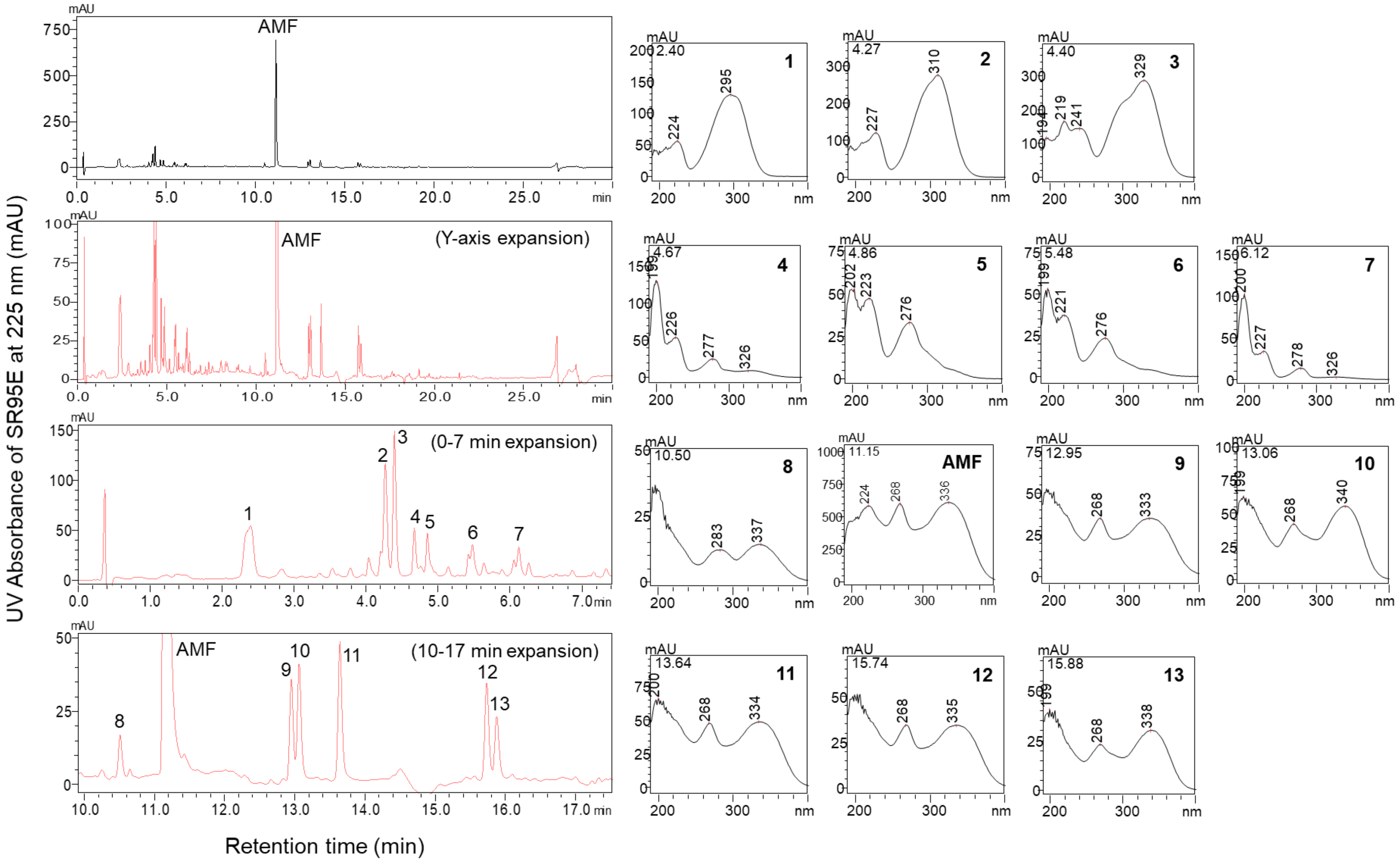

Figure S2. HPLC profiles of SR95E and UV spectra of its main phytochemical components detected at 225 nm. Concentration of samples: SR95E (5 mg/mL); Injection volume 5 µl.

2. Primer sequences used in this article are described in Table S1.

Table S1. Primer sequences used for qRT-PCR.

Gene (NCBI Reference Sequence)	Forward primer	Reverse primer
ACTIN (NM_001101)	GGCACCACACCTTCTACAAT	GCCTGGATAGCAACGTACAT
COL1A1 (NM_000088)	TGGCCTCGGAGGAAACTTT	GCTTCCCCATCATCTCCATTC
COL1A2 (NM_000089)	CGGTGGTGGTTATGACTTTGGT	GAAGGGTCTCAATCTGGTTGTTG
MMP-1 (NM_001145938)	AACACATCTGACCTACAGGATTGAAA	CTTGGTGAATGTCAGAGGTGTGA
MMP-2 (NM_001127891)	ACTGGAGCAAAAACAAGAAGACATAC	TCCATTTTCTTCTTCACCTCATTG
MMP-3 (NM_002422)	ATGCAGAAGTTCCTTGGATTGG	GATGCCAGGAAAGGTTCTGAAG
MMP-9 (NM_004994)	GCGCTGGGCTTAGATCATTC	GTGCCGGATGCCATTCA

Reference

25. Yu, S.; Yan, H.; Zhang, L.; Shan, M.; Chen, P.; Ding, A.; Li, S.F.Y. A review on the phytochemistry, pharmacology, and pharmacokinetics of amentoflavone, a naturally-occurring biflavonoid. *Molecules* **2017**, 22, 299.
